# Parental perspectives on human papillomavirus (HPV) vaccination of school-aged girls in Ilishan, Nigeria: A qualitative study protocol

**DOI:** 10.1371/journal.pone.0341563

**Published:** 2026-02-12

**Authors:** Aromoke Sanjo-Odutayo, Patrick Oyibo, Oghenowede Eyawo

**Affiliations:** 1 School of Nursing, Department of Community Health Nursing, Babcock University, Ilishan-Remo, Ogun State, Nigeria; 2 Department of Global, Public and Population Health, and Policy, School of Health and Medical Sciences, City St George’s, University of London, London, United Kingdom; 3 Department of Community Medicine, Faculty of Clinical Sciences, College of Health Sciences, Delta State University, Abraka, Nigeria; 4 School of Global Health, Faculty of Health, York University, Toronto, Canada; Federal University Otuoke, NIGERIA

## Abstract

The introduction of the human papillomavirus (HPV) vaccine in Nigeria in October 2023 aimed to vaccinate over 16 million adolescent girls through mass vaccination campaigns in schools, communities, and public places. Despite this initiative, many girls remain unvaccinated, particularly in some of the states included in the first phase of the rollout. Little is known about the perspectives of parents and caregivers who consent on behalf of their daughters regarding the HPV vaccine. This paper presents a qualitative study protocol and research design to explore what informed parents and caregivers’ acceptance or refusal of the HPV vaccine for their adolescent daughters during the campaign. Semi-structured in-depth interviews will be conducted with parents and caregivers of girls aged 9–14 years, attending both private and government primary and secondary schools in Ilishan, Ogun State, Nigeria. Participants will be purposively recruited from schools where the mass HPV vaccination campaign was implemented, with a focus on parents and caregivers who provide consent to take part in the study, regardless of whether they accepted or refused the HPV vaccination for their daughters when it was offered. Approximately 30 parents and caregivers will be interviewed until data saturation is reached and no new themes emerge from the data. Interviews will be transcribed verbatim and, where necessary, translated. Data will be coded and analysed thematically in an iterative process, allowing for the identification of key themes and patterns. This study aims to provide valuable insights into how parents perceive the HPV vaccine and the factors that informed their vaccination decisions. Findings will contribute to the development of context- and culturally-sensitive vaccination strategies, enhancing the success of HPV vaccination campaigns and supporting the global effort to eliminate cervical cancer**.**

## Introduction

Cervical cancer remains a significant public health challenge globally, with an estimated 662,044 new cases and 348,709 deaths reported in 2022 [1,2]. The highest incidence and mortality rates are observed in sub-Saharan Africa, South America, and South-East Asia [1,3]. The human papillomavirus (HPV) serotypes 16 and 18 are the primary causes of cervical cancer [4]. HPV is the most common sexually transmitted infection, with over 50% of sexually active women at risk of at least one serotype in their lifetime, with most contracting it after their sexual debut [5,6]. In Nigeria, cervical cancer incidence is alarmingly high, with a rate of 26.5 per 100,000 women, ranking it as the second most common cancer and the leading cause of cancer-related deaths among women aged 15–44 years, with an estimated 13,676 new cases and 7,000 deaths annually [7].

Prophylactic HPV vaccine has demonstrated the ability to prevent up to 90% of HPV-related cancers, including cervical, anal, oropharyngeal, vaginal, penile and vulvar cancers [8–10]. The HPV vaccine, introduced in 2006, was recommended by the World Health Organisation (WHO) in 2009 for early vaccination of pre-teen girls before they become sexually active, and been shown to prevent at least 70% of cervical cancer cases [7,11]. Since its introduction, over 100 million girls in the world have been vaccinated globally, with 95% of recipients residing in high-income countries [12]. The global strategy to eliminate cervical cancer aims to vaccinate 90% girls by age 15 years, screen 70% of women with a high-performance test by age 35 years and again by age 45 years, and ensure that 90% of women identified with cervical disease receive treatment [12]. The 90-70-90 target inspires optimism in low- and middle-income countries (LMIC) like Nigeria.

Given the proven efficacy and safety of HPV vaccine [13,14], and in response to the global call for cervical cancer prevention, Nigeria launched a nationwide free HPV vaccination campaign (using the Gardasil brand of HPV vaccine) in October 2023. The initiative aims to vaccinate over 16 million school-aged girls by 2025 through mass administration of the vaccine in schools and communities [15]. While national data on the number of vaccinated girls is scarce, parental approval remains crucial for the success of the program. Evidence from pre-launch surveys in Nigeria suggest that many parents and caregivers of school-aged girls are willing to consent to the use of the HPV vaccine if offered free of charge [16,17]. However, misinformation and concerns about vaccine safety persists among certain groups of the population [18]. Early findings since the vaccine launch indicate that parents and caregivers with higher educational levels and better knowledge of the HPV vaccine are more likely to consent to vaccination for their daughters [19]. Yet, there is limited understanding of how parents and caregivers personally perceive the HPV vaccine and how their responses shape the success of the vaccination campaign. This study aims to explore the perspectives of parents and caregivers in Ilishan Town, Nigeria, regarding the HPV vaccination of their daughters, providing valuable insights for culturally sensitive interventions and broader vaccine uptake strategies to support the global strategy to eliminate cervical cancer.

## Research aim

The aim of the study protocol is to describe the methodological approach for exploring the responses of parents and caregivers in Ilishan, Nigeria, to the free HPV vaccination program for their school-aged daughters.

### Research questions

1. What do parents and caregivers of school-aged girls in Ilishan know about HPV and the free one-dose HPV vaccination programme?
2. What does HPV vaccine mean to parents and caregivers of school-aged girls in Ilishan?
3. What and who are the sources of vaccination information for these parents and caregivers?
4. How do parents and caregivers respond to the offer of the free one-dose HPV vaccination for their girls?
5. What factors inform parents’ and caregivers’ responses, and why?

## Methods

### Study design

A qualitative descriptive design with an interpretative perspective will be employed to explore the experiences of parents and caregivers of school-aged regarding the free HPV vaccination. The design recognises that individual perceptions and experiences vary, allowing for rich, nuanced descriptions of parents’ and caregivers’ responses to the vaccine [20], depending on their individual perception [21]. The study will adopt an interpretative approach to analyse these descriptions, acknowledging the personal and contextual nature of decision-making in vaccination [22]. Ethical considerations will be integrated at every stage of the study, including in its design and reporting, ensuring the rights and confidentiality of participants [23].

### Ethics

The study received ethical approval from the Babcock University Health Research Ethics Committee (Ref. No: BUHREC 101624).

### Study setting

The study will be conducted in private and government primary and secondary schools in Ilishan, Ikenne Local Government Area, Ogun State, Nigeria. Participants will include parents and caregivers who provided consent during the October 2023 mass vaccination campaign for HPV vaccination of adolescent school-girls.

### Participants

Eligible participants will be parents and caregivers of girls aged 9–14 years at the time of the vaccination campaign in 2023. Participants must be fluent in English or Yoruba. We will recruit and focus on participants who provide consent to take part in the study, regardless of whether they accepted or refused the HPV vaccination for their daughters when it was offered. Recruitment is anticipated to begin in November 2025, following Local Government clearance, which will authorize access to the target population.

Participants will be identified through school registers and with the support of community gatekeepers, including teachers, registered nurses, and community health extension workers at Ilishan Primary Healthcare Centre. The researcher, accompanied by healthcare staff, will contact prospective participants via phone or home/workplace visits, provide an information sheet, and follow up within two days to confirm participation. Those who consent will be scheduled for one-on-one in-depth interviews at the Primary Healthcare Centre. The one-on-one interview approach allows participants to “give voice” to a range of issues surrounding HPV vaccination, providing detailed insights into parental decision-making, including how the vaccine is perceived and the factors influencing acceptance or refusal [24].

### Sampling strategy

A purposive sampling strategy will be employed to recruit approximately 30 parents and caregivers for interview, with data collection continuing until saturation is reached and no new themes emerge. This sample size is considered appropriate for qualitative research on the same issue among people of diverse backgrounds [25,26].

### Data collection

Data (i.e., perspectives of parents and caregivers) will be collected through semi-structured in-depth interviews lasting approximately 45 minutes. A tailored interview guide will be developed to explore participants’ knowledge, perceptions, and decision-making regarding the HPV vaccine. The interview guide will be piloted with a parent to ensure the relevance of the research questions [24] and the feasibility of the interview process in terms of time management during the interviews. Data collection is expected to take six weeks starting December 2025 to January 2026, following receipt of all local permissions.

Given the interpretative nature of this qualitative study, reflexivity will be actively incorporated throughout data collection and analysis to mitigate potential interviewer bias and enhance methodological rigour. We will maintain a reflexivity journal to document preconceptions, positionality, and evolving reflections arising during interviews and analysis. These reflections will be used to critically examine how researchers’ perspectives may influence the interview process and interpretation of the data. In addition, peer debriefing discussions will be conducted within the research team to critically examine emerging codes and themes, challenge assumptions, and strengthen analytic transparency. Researchers’ positionality and potential influences on data generation and thematic development will be explicitly acknowledged during analysis and reporting.

### Data analysis

Data will be analysed using a framework approach, which is not bound by a particular epistemological position and is similar to thematic analysis, being largely independent of theory [23,24]. This approach will be applied to the contextual data collected in the study, offering flexibility in managing a relatively large dataset and compatibility with NVivo 14 software. The framework approach is particularly suited to word-for-word data from in-depth interviews [23]. Its matrix-based format allows for systematic comparison both within and across cases, facilitating detailed thematic analysis while preserving the integrity of the raw data. Using this approach, all relevant aspects of parents’ and caregivers’ experiences will be captured. NVivo 14 software will be used to support coding and identification of key themes. Analysis of qualitative data will be conducted concurrently with data collection, allowing for iterative refinement of codes and emerging themes. We anticipate completing all analysis by February 2026.

### Informed consent

Participants will be provided with a detailed information sheet in English, with a Yoruba translation available for those who primarily comprehend the local language. The information sheet will outline the purpose of the study, procedures, the nature of the interview, how data will be managed, and the potential risks and benefits of participating in the study. Written or oral informed consent will be obtained, with oral consent recorded when written consent is not feasible. All collected data will be treated as confidential and stored securely on encrypted, password protected devices, with access limited to authorised research personnel only.

## Discussion

The findings from this study will provide valuable insight into how parents and caregivers of school-aged girls in Ilishan perceive the HPV vaccine and the factors that informed the vaccination decision for their daughters. Also, gathering first-person information of consenting parents and caregivers will shed more light into parents’ approach to disease prevention practices and protection for young girls. This understanding will be essential for public health experts and vaccinators in developing targeted public health interventions that address parental concerns, enhance information around vaccines, and ultimately, improve vaccine uptake. By considering the contextual factors that inform vaccination decisions, the study will contribute to the global effort to eliminate cervical cancer and promote the health and well-being of adolescent girls in Nigeria and similar settings. More broadly, it will provide additional contextual understanding that can help increase confidence in vaccination in general.

## Limitations

The focus of this study on English- or Yoruba-speaking parents in Ilishan limits its generalizability to other regions or linguistic groups in Nigeria. However, given the demographic characteristics of the community, the findings are expected to be transferable to similar rural settings in the country. In addition, the purposive sampling strategy, which relies on school registers and contact through community gatekeepers, may inadvertently exclude certain parental perspectives, such as parents who are not accessible through schools, lack reliable telephone access, or are less engaged with formal educational or health systems. This may further limit the range of perspectives captured and should be considered when interpreting the findings. Additionally, some cultural nuances or expressions may be lost during translation from English to local language and vice versa. To mitigate this, the researcher will conduct transcription and translation carefully and include untranslatable words in parentheses.

## Dissemination

We anticipate that results will be ready for publication by March 2026. Findings will be disseminated widely through publication in an academic journal, conference presentations, and a detailed report to research sponsors as well as to Ikenne Local Government, where parents and caregivers in Ilishan will be recruited. To ensure accessibility for local stakeholders, findings will also be shared through plain-language summary reports and brief presentations tailored to community and local government audiences. Participant quotes will be included where appropriate to illustrate key findings, while ensuring that privacy and confidentiality are fully protected. Results will additionally be shared through social media platforms, such as LinkedIn, X, and via blog posts to reach a broader audience. This multi-pronged dissemination strategy aims to maximize the visibility and impact of the study across academic, vaccination program, and health policy contexts, while ensuring that findings are accessible to the communities involved.

## Supporting information

S1 File PLOS ONE inclusivity questionnaire. (PDF)
